# Comparative Genomics of *Ralstonia solanacearum* Identifies Candidate Genes Associated with Cool Virulence

**DOI:** 10.3389/fpls.2017.01565

**Published:** 2017-09-13

**Authors:** Ana M. Bocsanczy, Jose C. Huguet-Tapia, David J. Norman

**Affiliations:** ^1^Mid-Florida Research and Education Center, Department of Plant Pathology, University of Florida, Apopka FL, United States; ^2^Department of Plant Pathology, University of Florida, Gainesville FL, United States

**Keywords:** transcriptional regulators, temperature, quorum-sensing, T3SS, T6SS, effectors

## Abstract

Strains of the *Ralstonia solanacearum* species complex in the phylotype IIB group are capable of causing Bacterial Wilt disease in potato and tomato at temperatures lower than 24°C. The capability of these strains to survive and to incite infection at temperatures colder than their normally tropical boundaries represents a threat to United States agriculture in temperate regions. In this work, we used a comparative genomics approach to identify orthologous genes linked to the lower temperature virulence phenotype. Six *R. solanacearum* cool virulent (CV) strains were compared to six strains non-pathogenic at low temperature (NPLT). CV strains can cause Bacterial Wilt symptoms at temperatures below 24°C, while NPLT cannot. Four *R. solanacearum* strains were sequenced for this work in order to complete the comparison. An orthologous genes comparison identified 44 genes present only in CV strains and 19 genes present only in NPLT strains. Gene annotation revealed a high percentage of genes compared with whole genomes in the transcriptional regulator and transport categories. A single nucleotide polymorphism (SNP) analysis identified 265 genes containing conserved non-synonymous SNPs in CV strains. Ten genes in the pathogenicity category were identified in this group. Comparisons of type 3 secretion system, type 6 secretion system (T6SS) clusters, and associated effectors did not indicate a correlation with the CV phenotype except for one T6SS VGR effector potentially associated with the CV phenotype. This is the first *R. solanacearum* genomic comparative analysis of multiple strains with different temperature related virulence. The candidate genes identified by this comparison are potential factors involved in virulence at low temperatures that need to be investigated. The high percentage of transcriptional regulators among the genes present only in CV strains supports the hypothesis that temperature dependent regulation of virulence genes explains the differential virulence phenotype at low temperatures. This comparison contributes to find new possible connections of temperature dependent virulence to the previously described complex regulatory system involving quorum-sensing, phenotype conversion (phcA), acyl-HSL production and responses to SA. It also added novel candidate T6SS effectors and useful detailed information about the T6SS in *R. solanacearum.*

## Introduction

*Ralstonia solanacearum* has been listed among the top 10 plant pathogenic bacteria because of its broad host range, genetic diversity, geographical spread, and the destructive symptoms it produces (Mansfield et al., 2012). This bacterial species can infect more than 200 plant species, causing different diseases including Brown Rot in potatoes, Bacterial Wilt of solanaceous plants and ornamentals, and Moko Disease of musaceae family (Hayward, 1964; Elphinstone, 2005). The bacterial species as a whole is heterogeneous and has been difficult to classify. Currently the most used classification divides the species into four main phylotypes, correlated with geographical locations in tropical, subtropical, and temperate regions of the world (Fegan and Prior, 2005; Remenant et al., 2010). *R. solanacearum* can survive in wet soil for years, from there it can infect plant roots, penetrating through wounds or natural openings (Buddenhagen and Kelman, 1964). Once in the plant, it moves to the xylem, where it multiplies to large population and produces the wilting symptoms. The disease is devastating. Its overall economic impact is difficult to quantify due to the geographical span of the disease and the degree and variety of symptoms it causes on the different crops. In the United States, the most significant economic impact is on potato, tomato, and tobacco production in southern states, caused by endemic strains. A particular subgroup of phylotype II populations, known as R3B2 or phylotype IIB-sequevar 1, can cause disease in potato and to a lesser degree in tomato at temperatures below 24°C (Ciampi and Sequeira, 1980; Milling et al., 2009). This monophyletic group of strains is not present in the United States, although it has been inadvertently introduced a number of times through imported geraniums, without establishment. Because of the threat this group represents for United States agriculture, it has been designated select agent under the United States Agricultural Bioterrorism Protection Act of 2002 (Lambert, 2002). Although R3B2 strains are not present in the United States, CV strains that belong to phylotype IIB but not in the R3B2 group and are pathogenic in tomato and potato plants at low temperatures are present in the United States and were isolated from pothos and other ornamentals (Norman and Yuen, 1997, 1999; Bocsanczy et al., 2012). P673, a representative of this group that was previously identified as Race 1 biovar 1, belongs to phylotype II-sequevar 4NPB (Bocsanczy et al., 2013) and has been recently identified in the Caribbean as an emergent group closely related to the Moko disease strains (Deberdt et al., 2014). The danger of introduction of R3B2 strains and the presence in the United States of other groups of *R. solanacearum* strains that are capable of causing disease at low temperatures highlights the need to understand the sources and mechanisms of pathogenicity at low temperatures. Advances in DNA sequencing technology, along with lower sequencing costs and computational tools development, have enabled the inference of phenotypic traits and functional genomics analysis through genomic comparisons (Jim et al., 2004; Feldbauer et al., 2015). Protein-coding genes in microbial genome databases are annotated by sequence similarity and conserved domains using Clusters of Orthologous Groups (COGs), protein families (Pfam), and other orthologous conserved domains databases across genomes (Sonnhammer et al., 1998; Tatusov et al., 2000; Punta et al., 2012; Tatusova et al., 2015). Protein functions can be inferred by phylogenetic profiling based on the absence or presence of orthologous genes in microbial genomes and similar profiles are thought to share similar functions (Pellegrini et al., 1999). Thus, the presence or absence of orthologous genes in microbial genomes is very informative, when combined with phenotype profiles, which group microbial genomes by the desired phenotype (Jim et al., 2004; Goh et al., 2006; Tamura and D’Haeseleer, 2008). It allows the prediction of metabolic functions (Kanehisa et al., 2014), protein–protein interactions (Szklarczyk et al., 2015), and particular traits, such as virulence at low temperature in our case. Based on the hypothesis that proteins can lose or alter functions when there are differences in amino acids, a genome-wide search for conserved alleles in particular genome groups associated with a particular trait can identify a group of genes that might be associated with the trait (Read and Massey, 2014). In addition, comparative examination of virulence associated systems such as T3SS and T6SS and associated sets of effectors (effectorome) could identify differences associated with the cool virulence trait. In order to simplify terminology, in this work we will refer to the strains capable of causing disease at low temperatures as CV strains and to the ones that are not capable of causing disease at low temperatures as NPLT.

Few comparative works of *R. solanacearum* strains with different virulence at low temperature have been published. We reported differential proteomics profiles of CV and NPLT strains at 30 and 18°C when in contact with plant roots (Bocsanczy et al., 2014a). In that work, we compared protein expression of two CV with two NPLT *R. solanacearum* strains. We showed that 101 proteins at low temperature were downregulated in NPLT strains while their expression was unchanged or upregulated in CV strains. In that case, the proteins were present in all the strains and the difference was in their expression, leading to the hypothesis that the difference in virulence might be partially explained by the differential regulation of virulence associated genes. Another work examined the difference between the transcriptome of one CV strain and one NPLT either in rich media or *in planta* during infection (Meng et al., 2015). In this work the strains had different transcriptional responses to temperature change. At cool temperature the CV strain upregulated a cluster encoding a quorum sensing-dependent and a hypothetical proteins. These genes were not present in the NPLT strain, and were shown to contribute to virulence of CV at low temperature leading to the hypothesis that genomic differences between CV and NLPT strains also may contribute to differential virulence at low temperature.

In the present work, we hypothesize that candidate genes which contribute to the CV phenotype can be identified by genotypic differences either by the absence/presence of genes or SNP differences correlated with the phenotype. We present the first genomic comparison of 12 *R. solanacearum* genomes with the aim to identify and characterize *in silico* protein-coding genes associated with the cool virulence phenotype. To date many genomes of *R. solanacearum* have been sequenced and are publicly available either complete or as whole genome shotguns (WGS) in the NCBI database, however, there is limited information about pathogenicity at low temperatures. We selected six CV and six NPLT strains for this comparison. We generated draft genomes sequences of four strains reported in this work to complete this comparison. Overall, only 44 orthologous protein-coding genes are present exclusively in CV strains and only 19 are present exclusively in NPLT strains. Annotation distribution analysis of the 44 orthologous genes revealed a high percentage of transcriptional regulators and transport categories, compared with their abundance in complete genomes, and absence of virulence-associated genes. SNP comparison identified 265 non-synonymous SNPs that correlate with the cool virulence phenotype. Comparative analysis of two secretion systems revealed a high degree of core genes conservation among strains, and a high degree of diversity for their associated effectorome.

## Materials and Methods

### *R. solanacearum* Strains

Twelve strains were selected for this study. **Table 1** lists the strains, their source, the host, their phylogenetic classification, the GenBank sequence accession number, and the genome public references.

**Table 1:** Strains of *Ralstonia solanacearum* used for the genome comparison.

Strain (other names)	Origin	Isolated	Phylotype/sequevar	Cool virulence	Genome reference
P742 (GMI1000)	French Guyana	Tomato	I/18	NPLT	Salanoubat et al., 2002
P597	United States	Tomato	IIA/38	NPLT	This work
P660 (K60-1)	United States	Tomato	IIA/7	NPLT	Remenant et al., 2012
P795 (CIP120)	Peru	Potato	IIA/38	NPLT	This work
P796 (CFBP2957)	French West Indies	Tomato	IIA/36	NPLT	Remenant et al., 2010
P673	United States	Pothos	IIB/4-NPB	CV	Bocsanczy et al., 2013
P714 (UW551)	Kenya	Geranium	IIB/1	CV	Gabriel et al., 2006
P797 (CFBP6783)	Martinique	Heliconia	IIB/4-NPB	CV	CIRAD and this work
P800 (UW163)	Peru	Plantain	IIB/4	CV	CIRAD
P807 (23-10BR)	Brazil	Potato	IIB/27	CV	Clarke et al., 2015
NCPPB909	Egypt	Potato	IIB/1	CV	Yuan et al., 2015
P799 (CFBP3059)	Burkina Faso	Eggplant	III/48	CV	This work

### Pathogenicity Tests

Pathogenicity tests were carried out as described in previous works (Bocsanczy et al., 2012) with small modifications. Briefly, bacteria were grown for 48 h on Nutrient Agar (NA) plates. Bacterial colonies were diluted in saline solution (NaCl at 8.5 g/liter) up to OD600 = 0.01 (1 × 10^7^ CFU/ml) using a spectrophotometer (Nanodrop 2000C, Thermo Scientific). Individual plants were inoculated with 50 ml of the dilution, poured into the pot over the soil, and placed in temperature, light, and humidity controlled chambers. Temperature was set at 18°C. At the end of 30 days, wilted plants were counted, and the percentage of wilted plants was calculated. The experiment was repeated two times with at least 10 plants each treatment.

### Genome Sequencing

*Ralstonia solanacearum* strains were grown at 28°C overnight in CPG (Casamino acid-peptone-glucose) medium to OD_600_ = 1.2. Genomic DNA was obtained using Ultraclean Microbial DNA Isolation Kit cat# 12224-50 (Mobio, Carlsbad, CA, United States) following manufacturer’s instructions. Libraries and sequencing were performed using the Illumina HiSeq-2500 with pair end 100 cycles with 150X coverage. Reads were processed in house with Geneious 9.0.2^1^ (Kearse et al., 2012). Adapter and low quality sequences at the reads ends were trimmed. Reads shorter than 50 nt or of low quality were discarded. The remaining reads were assembled with Geneious. The contigs were checked for remaining adaptor sequences and low quality nucleotides. Contigs less than 200 nt were discarded.

### Genome Assemblies and Annotation

Contigs were assembled against closely related reference genomes, using Geneious software. Then contigs were sorted and numbered in order with the assembly. Contig annotation was performed with the PGAP of NCBI. Manual editing of annotation was performed with Artemis (Carver et al., 2012).

### Genome Statistics

#### Quality Assemble Tests

Assessment of genome assembly completeness was conducted using Benchmarking Universal Single-Copy Orthologs (BUSCO v3) (Simao et al., 2015). Dataset corresponding to betaproteobacteria was used to evaluate the assembly completeness and gene prediction of the assembled contigs for each genome. In addition SNPs were verified by re-mapping raw reads against the assembled contigs of each genome using Bowtie 2 (Langmead and Salzberg, 2012). Mapping files were transformed into binary files using SAMtools (Li et al., 2009) and the output was used to obtain a corrected set of contigs using Pilon tool v1.16 (Walker et al., 2014).

#### Significance of Comparative Results

A shuffling experiment was performed in order to test the significance of the comparative genomics comparison. Each one of the 12 strains in the comparison was randomly assigned to an arbitrary class (1 or 2) using a random generator function. Then the strains were searched for genes present or absent in each class and for genome-wide SNP linked to each class. This procedure was repeated 10 times.

### Genome Comparison

#### Orthologous Genes and Pan-Genome

Fasta files and GenBank annotated contigs files were concatenated in one pseudomolecule for visualization and comparative analysis. Both molecules of each published complete genome were also concatenated to facilitate comparison.

Compared strains were classified as “CV” and “NPLT” based on their ability to cause disease at 18°C. Pseudomolecules were aligned with MAUVE progressive alignment tool (Darling et al., 2004) using minimum identity at 60% and minimum coverage at 70% as criteria. Orthologous gene list as calculated by MAUVE was used to produce gene distribution, pan-genome, and identify orthologous genes present only in one of the two phenotypes. Presence and absence of genes were confirmed by protein sequences BLASTP searches (Altschul et al., 1997).

Prokaryotic genome annotation pipeline functional annotation of selected groups of genes were updated and confirmed with annotation with Blast2GO software (Conesa et al., 2005).

### SNP Detection and Comparison

Single nucleotide polymorphisms detection was carried out with kSNP3 v3 software (Gardner et al., 2015). Pseudomolecules of compared genomes were used as input using as a reference the UW551 genome. KSNP3 v3 finds SNP positions in genomes using MUMmer (Delcher et al., 2002) and produces clusters of SNPs by genome groups. Optimal kmer length (21 bp) for SNP detection was selected using the Kchooser script implemented in the KSNP tool. The output is summarized in a variant call format file (VCF) file that was analyzed and filtered for those SNPs that are conserved only in the CV strains. SNPdat software was used to annotate SNPs (Doran and Creevey, 2013). Additional visual inspection of SNPs and analysis of amino acid conservation in selected cluster of orthologs was conducted using Jalview alignment tool (Waterhouse et al., 2009).

#### PCR Amplification of Transcriptional Regulators

Conserved primers were designed to amplify the complete CDS of the transcriptional regulators found in the comparison. Transcriptional regulators P673_16370 and P673_16375 are adjacent, and they were amplified together with one pair of primers. Primer sequences are listed in Supplementary Table S1A, and specific PCR conditions such as annealing temperatures and extension time in Supplementary Table S1B. Some of the reactions required DMSO 3% since products are high in GC contents. General PCR conditions were: [denaturation: 98°C 30 s; amplification: 25x (98°C 15 s, annealing 30 s, extension time min); final extension: 72°C 10 min]. 0.8% agarose gels were prepared and run at 90 V for 1 h in order to visualize PCR bands.

#### Clusters and Effectors Comparisons

Individual clusters of T3SS and T6SS were compared, organized, and manually annotated with aid of MAUVE, Artemis, and Geneious software. T3SS effectors were predicted using the RalstoT3E tool (Peeters et al., 2013). T3SS effectors were identified *in silico* using the software tool Secret6 (Li et al., 2015), and extracting viral genome replication (VGR), proline-alanine-alanine-arginine (PAAR), and rearrangement hot spots (Rhs) domains from gene annotations. All predicted sequences were manually revised and checked for frameshifts or truncations.

#### Supporting Data

Genome sequences of all compared genomes are in the NCBI database under accession numbers: GMI1000 [NC_003295/NC_003296], P597 [JIBY00000000], K60-1 [CAGT01000001], P795 [JXAY00000000], CFBP2957 [NC_014307], P673 [JALO00000000], UW551 [AAKL00000000], P797 [JXAZ00000000], UW163 [CDMB00000000], 23-10BR [JQOI00000000], NCPPB909 [JNGD00000000], and P799 [JXBA00000000]. K60-1 megaplasmid sequence is available at Microscope Genome annotation and Analysis Platform.^2^

Raw sequence data was deposited in the NCBI Sequence Read Archive (SRA) under accession numbers: P597 [experiment: SRX2850877, RUN: SRR5595754], P799 [experiment: SRX2849979, RUN: SRR5593154], P797 [experiment: SRX2849980, RUN: SRR5593153], P795 [experiment: SRX2849981, RUN: SRR5593152], and P673 [experiments: SRX2850875 (Illumina), SRX2850876 (LS454), RUNS: SRR5595753 (Illumina), SRR5595752 (LS454)].

## Results and Discussion

### Selection of *R. solanacearum* Strains for the Comparison

**FIGURE 1:**
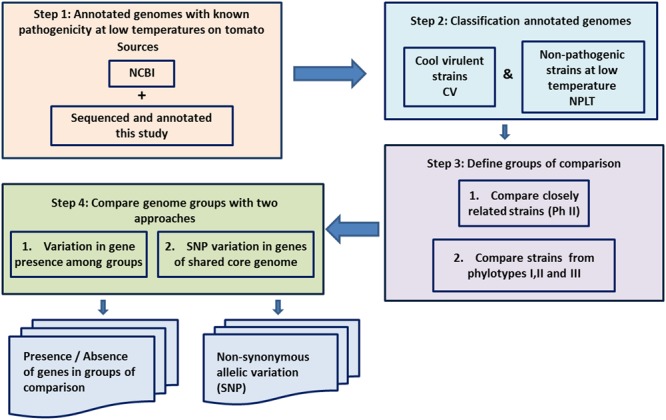
Flowchart of the temperature dependent gene comparative approach. Conceptual pipeline showing how to enrich the list of potential genes associated with virulence at low temperature. Step 1: Compile annotated genome sequences that have been tested for pathogenicity between 18 and 24°C on tomato plants. Step 2: Classify genome sequences into either CV or NPLT. Step 3: Define groups of comparison based in several criteria. Step 4: Compare genome groups with several approaches.

**FIGURE 2:**
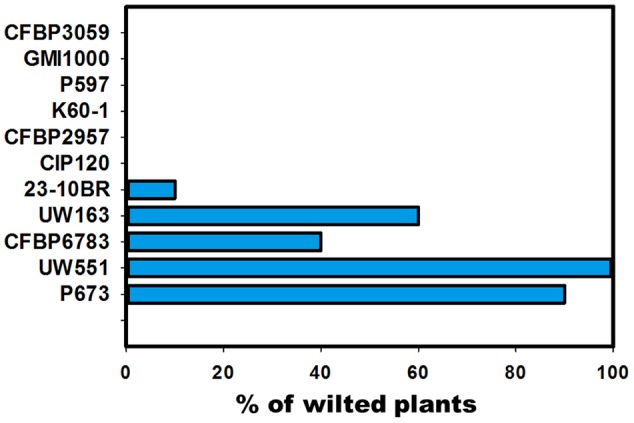
Pathogenicity test at 18°C. Ten to fifteen tomato plants per strain were drench inoculated with 50 ml of a 10^7^ CFU/ml bacterial suspension. At the end of 30 days incubation period in temperature, light, and humidity controlled chambers, the number of wilted plants were counted. The histogram shows the % of wilted plants at the end of the experiment. Experiments were repeated two times. Strain NCPP909 was not available for testing.

Twelve strains of *R. solanacearum* were selected for the genomic comparison (**Table 1**). Equal number of CV strains and NPLT strains from the phylotypes I, II and III, with tomato as the common host were compared. The process flowchart is described in **Figure 1**. The first step was to obtain the annotated genomes for the strains to be compared. Four strains were sequenced to complete the genome database necessary for the comparison. The second step was to confirm their pathogenicity phenotype at low temperature. Since there is little information about cool virulence in the literature, besides the known low temperature pathogenicity of the R3B2 group, candidate strains were tested for low temperature pathogenicity. Strains P673, CFBP6783, UW551, 23-BR10, and UW163 were pathogenic at 18°C, while P597, CIP120, K60-1, CFBP2957, GMI1000, and CFBP3099 were non-pathogenic (**Figure 2**). We used previous reports of cool virulence for strain NCPPB909 since we did not have the strain available for testing (Clarke et al., 2015; Yuan et al., 2015). Genomes of strains GMI1000, K60-1, CFBP2957, P673, UW551, UW163, 23-BR10, and NCPPB909 were publicly available at the time of this work (Salanoubat et al., 2002; Gabriel et al., 2006; Remenant et al., 2010, 2012; Bocsanczy and Norman, 2013).

### General Features of Genome Sequences

The genomes of P597, P795 (CIP120), P797 (CFBP6783), and P799 (CFBP3059) strains were sequenced for this work on Illumina HiSeq platform generating paired end sequences. The reads were assessed, processed, and assembled with Geneious 9.0.2 (Kearse et al., 2012). The genomes were assembled in contigs and aligned to the most similar complete genome publicly available as reference. Contigs were concatenated to form pseudomolecules following the reference order. Assembly statistics are shown in Supplementary Table S2. Reads and assemble quality was measured by several metrics. The Phred score which provides a measurement of the reads quality (Ewing and Green, 1998; Ewing et al., 1998) was approximately 36 for all the strains. A Phred score of 20 or above is considered acceptable, thus 36 corresponds to accurate sequences (Illumina, 2011). The read depth is the number of reads that include a given nucleotide. It is measured as number of times the length of the genome is covered by the length of reads and gives a sense of the sequencing coverage. Average coverage ranged between 140X and 252X. A coverage of 150X or more is usually considered good coverage with Illumina platform for whole genome sequences (Desai et al., 2013). Another important measure of quality is the N50 length. This metric defines the shortest sequence length of the set of contigs ordered from longest to shortest for which the combined length represents at least 50% of the assembly (Miller et al., 2010). The longer N50, the better the assembly provided high genome coverage is achieved (Desai et al., 2013). P597 and P799 assemblies had a much lower N50 value than P797 and P795. As observed in Supplementary Table S2, the number of contigs mapped for P597 was 530 and for P799 was 399 compared with 145 for P795 and 178 for P797. Assessment of genome assembly completeness indicates that the genomes used in this study contain in average 97% of betaproteobacteria core genome reported in the BUSCO database. The lowest set of completed genes was found in UW551 with 93% of the total set in BUSCO database. Within the group of genomes we assembled in this study, the average increased to 98%. Our results indicate that although some genes are missing we believe that the level of completeness can offer a fair approximation of the gene content of our selected sample (Supplementary Table S3A).

A summary of general genomic features of the four *R. solanacearum* strains sequenced is presented in **Table 2**. The genomes sequenced had similar lengths and were organized in the chromosome and megaplasmid similarly to other *R. solanacearum* strains, confirming that the sequencing covered most of the genomes and did not have long missing sections. Their estimated average size was approximately 5.6 Mb, including a chromosome of approximately 3.5 Mb and a megaplasmid of approximately 2.1 Mb (**Table 2**). Differences between distributions in chromosome and megaplasmid might be due to misplacements of contigs in the draft genomes. The number of proteins predicted ranges between approximately 4500 and 4900. The number of predicted pseudogenes ranges between 141 and 216. Differences in these numbers with other publicly available strains are explained by different bioinformatics methods used to predict open reading frames (ORFs) and differences in quality of the draft genomes.

**Table 2:** General features of the genomes sequenced for this work.

Strain	WGS (contigs)	Genome size (Kbp)	Chromosome (Kbp)	Megaplasmid (Kbp)	G+C content (%)	# of CDS	# of RNAs	# of pseudogenes
P797	178	5,557	3,568	1,989	66.64	4677	57	146
P597	530	5,648	3,296	2,352	66.45	4580	57	206
P795	145	5,671	3,551	2,120	66.56	4776	57	141
P799	399	5,396	3,391	2,005	66.90	4573	58	216

### Genome Comparisons and Genes Distribution

Genome concatenated pseudomolecules were compared using MAUVE software (Darling et al., 2004). In a first step as described in the flowchart (**Figure 1**, Step 3), we included six CV strains all belonging to phylotype IIB and four NPLT strains belonging to phylotype IIA (results not shown). All CV strains we have currently identified as CV belong to phylotype IIB sequevars 1, 4, and 27. We included strain UW163, whose main host is Musa (banana). UW163 is also capable of causing disease in tomato at both 30 and 18°C. We also included strain 23-10BR, an atypical R3B2 strain that was classified as sequevar 27 due to its differences with the usually clonal R3B2 strains (Clarke et al., 2015). In order to eliminate orthologous genes that might be present in NPLT strains due to phylogenetic closeness, but not related to the phenotype, for our definitive comparison we added a strain from phylotype I (GMI1000) and a strain from phylotype III (P799-CFBP3059) which are NPLT completing a 12 strain comparison. The phylogenetic relationship of the strains compared in this work are illustrated in Supplementary Figure S1.

**FIGURE 3:**
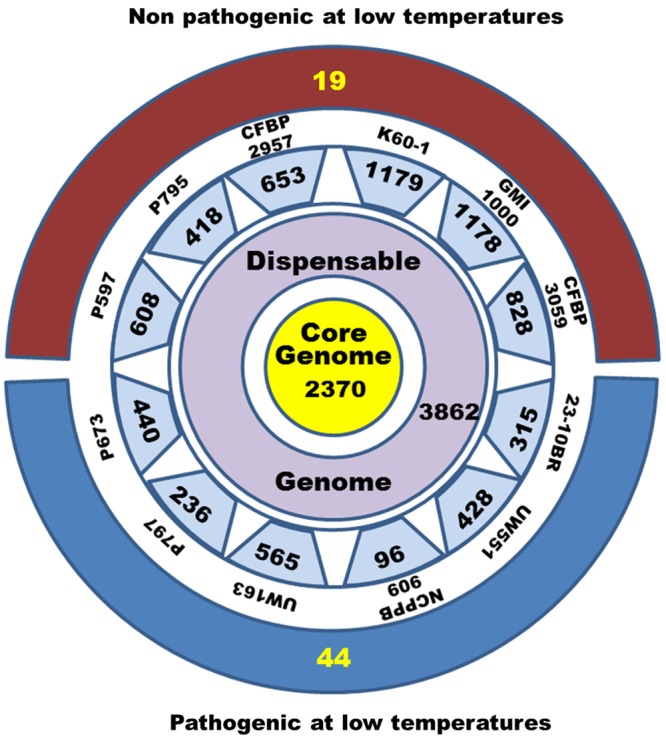
Distribution of gene numbers in the pan-genome of the compared *Ralstonia solanacearum* strains. Twelve strain genomic sequences were aligned and orthologous genes were identified using Mauve software. The core genome (yellow) contains all the common genes of the 12 strains. The dispensable genome contains all the genes common from 2 to 11 strains. The number of unique genes are indicated in individual blue boxes with the strain name. The external red bar indicates the number of genes (19) that are present only in non-pathogenic strains at low temperatures and the external blue bar indicates the number of genes (44) common only to CV strains.

Two approaches were used to extract lists of genes correlated with the CV phenotype. (1) Phylogenetic profile grouping genes present or absent in each phenotypic group; and (2) Genome-wide search of non-synonymous SNP associated with each phenotypic group (**Figure 1**, Step 4). The core genome of the 12 strains comparison had 2370 genes (**Figure 3**). The number of genes unique to a strain varied from 96 for NCPPB909 to 1179 for K60-1. The variation in number of unique genes is probably due to differences in annotation systems and the quality of the draft genomes. A better sequence coverage will have more genes that may be missing due to sequencing gaps in other genomes. Annotation is also important. If the CDS prediction and databases searched are different, there might be a decrease in the number of orthologs detected. An example is found with strain K60-1. The sequence was taken from the Microbial Genome Annotation and Analysis Platform (MicroScope) (Vallenet et al., 2013) database because the megaplasmid sequence was missing from the EMBL and NCBI databases. The different prediction methods used for CDS probably caused differences in the number and location of CDS produced. The list of the 44 orthologous genes present only in CV is listed in Supplementary Table S4A, while the 19 present only in NPLT strains is listed in Supplementary Table S4B.

In order to determine if the observed association between genotype and phenotype is different from expected associations by chance to two arbitrary categories, we performed a shuffling experiment. Each strain was assigned randomly to either arbitrary category 1 or 2, and the set of genes present or absent in each category was calculated. We repeated this experiment 10 times. As observed in Supplementary Table S3B only two of the shuffling experiments produced a set of genes. Experiment 5 produced a set of five genes present only in category 1 and Experiment 8 a set of four genes present only in category 2. The distribution of these genes were completely atypical. We confirmed that the correlation between phenotype and genotype for CV and NPLT strains was not random.

**FIGURE 4:**
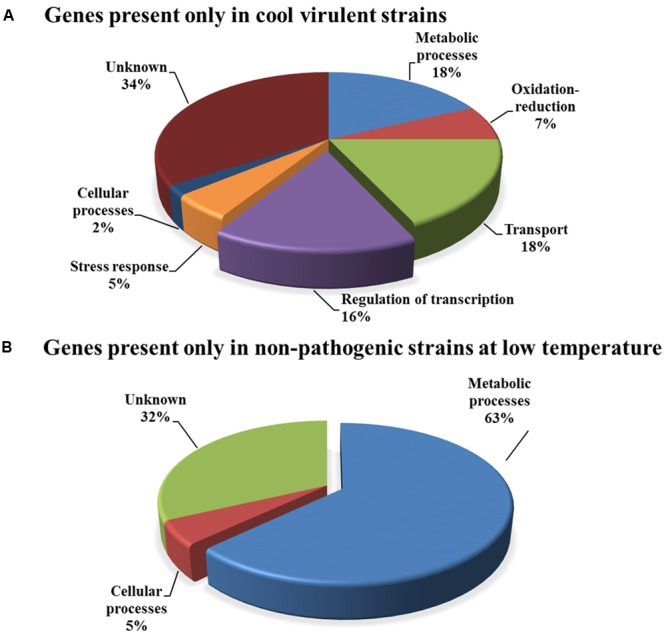
Distribution by biological process categories of orthologous genes associated with the temperature virulence phenotype. Annotated genes were classified according to biological process Gene Ontology (GO) terms. **(A)** Genes present only in CV strains. **(B)** Genes present only in non-pathogenic strains at low temperature.

Genes were annotated using Gene Ontology (GO) terminology for biological process categories (Ashburner et al., 2000). The genes distribution by general biological processes is shown in **Figure 4**. In the group of genes present only in CV strains (**Figure 4A**), the highest percentage category was 34% for unknown genes, as expected. Interestingly, the percentages of categories for transport and regulation of transcription were higher than expected in a whole genome distribution, with 18 and 16%, respectively. The transport category is usually in the range of 10–14% and regulation of transcription is typically in the range of 6% for *R*. *solanacearum* and other similar bacterial genomes (Madeira and Gabriel, 2007). Particularly, the regulation of transcription category is of interest because its high number supports the hypothesis that differential regulation of virulence genes largely explains the different virulence between CV and NPLT strains (Bocsanczy et al., 2014a). The absence of virulence genes in this list is also indicative that there are no absent virulence genes only in NPLT strains. The distribution of genes present only in NPLT strains was completely atypical (**Figure 4B**). Only three categories were represented where metabolic processes and unknowns were dominating with 63 and 32%, respectively. There were no genes in the categories transcriptional regulators or transport. Almost all genes in this comparison belonged to one cluster mostly containing genes associated with fatty acid and lipid metabolism. This explains the atypical functional distribution of the gene annotations.

### Transcriptional Regulators Present Only in the Compared CV Strains

**FIGURE 5:**
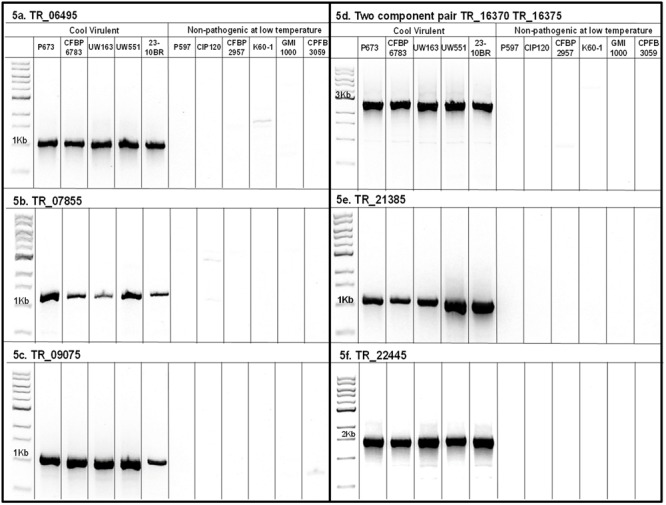
PCR amplification of seven transcriptional regulators found only in the CV strains in the comparison. PCR was run with primers described in Supplementary Table S1 at recommended annealing temperatures. PCR reactions were run in an agarose gel 0.8% 90 V for 1 h. The presence of bands indicates the presence and completeness of the genes in CV strains and their absence in NPLT strains.

Seven transcriptional regulators were identified as present only in CV strains. **Table 3** lists the locus tags of the regulators homologs for the six CV strains compared in this work. The list was manually curated in order to verify their presence or absence in CV or NPLT. An *in silico* analysis of these genes revealed that most were Lys type regulators. The LysR family of transcriptional regulators is the most abundant in bacterial genomes. This family is characterized by a conserved structure with a DNA-binding helix-turn-helix motif in their N-terminus. LysR are conserved global transcriptional regulators that can act as activators or repressors. They have evolved to regulate genes with diverse functions including nitrogen fixation, stress responses, toxin production motility, metabolism, quorum sensing, and virulence (Maddocks and Oyston, 2008). They did not seem to belong to a particular cluster or to occur in close proximity to known virulence factors. Supplementary Figure S2 illustrates the annotated adjacent genes of these transcriptional regulators. The sequences of genes RSP673_21385 and RSP673_22445 appeared to be truncated; this could be an artifact of the draft genomes assemblies. We designed primers in conserved regions at the N-terminus and C-terminus of each gene to confirm the presence/absence of complete transcriptional regulators in CV/NPLT strains, respectively, and to determine if they were truncated or not. Primers used and recommended annealing temperatures are shown in Supplementary Table S1. PCR amplification of the putative genes carried with the described primers confirmed that the transcriptional regulators are present only in the CV strains compared and are not present in the NPTL strains (**Figure 5**). Additionally, we confirmed that genes RSPP673_21385, RSP673_22445, and their homologs in P797 (CFBP6783) are not truncated as confirmed by the presence of bands of expected length in the PCR products.

**Table 3:** Orthologs of transcriptional regulators present only in CV strains.

P673	CFBP6783	UW551	NCPPB909	23-10BR	UW163
RSP673_06495	RSP797_06395	RRSL_02465	CQ06_06630	KR96_09045	UW163_RS10960
RSP673_07855	RSP797_07815	RRSL_04509	CQ06_14165	KR96_20550	UW163_RS15060
RSP673_09075	RSP797_08995	RRSL_00185	CQ06_01545	KR96_21300	UW163_RS13925
RSP673_16370	RSP797_19820	RRSL_00747	CQ06_21525	KR96_12215	UW163_RS16390
RSP673_16375	RSP797_19825	RRSL_00748	CQ06_21530	KR96_12220	UW163_RS16385
RSP673_21385	RSP797_22290	RRSL_00108	CQ06_07015	KR96_15245	UW163_RS19305
RSP673_22445	RSP797_23380	RRSL_00831	CQ06_05885	KR96_08010	UW163_RS20305

In order to strengthen our results we expanded our PCR test to strains of *R. solanacearum* that were not included in our genomic comparison because their genome has not been sequenced. We tested strains P446, P487, P618, and P666 previously shown to be CV (Bocsanczy et al., 2012) and strains P781, P822, P824, and UW757 which we confirmed were non-pathogenic at 18°C (results not shown), As shown in Supplementary Figure S3, the CV strains have all transcriptional regulators while the NPLT do not. Supplementary Figure S3 also show the phylogenetic classification of these strains. These transcriptional regulators are of special interest for further investigation because their presence supports the hypothesis that gene regulation contributes largely to the differences in virulence at low temperatures. This is consistent with the results of previous proteomics works where abundance of virulence and virulence associated proteins was different for CV strains than for NPLT strains at low temperatures (Bocsanczy et al., 2014a).

### SNP Comparison

The list of SNPs for all the strains compared using UW551 as reference were produced in a VCF. The SNPs were verified by re-mapping the raw reads available to the contigs with only few differences encountered. kSNP3 was ran with different k-mer sizes obtaining the optimum run with a k-mer size of 21 producing a 98.18% of SNPs aligned only one time (non-ambiguous alignment). VCF file was parsed and filtered to select only the SNPs that were conserved in the CV strains. Then the position of the selected SNPs was overlapped to the annotation file and only the SNPs in gene sequences producing changes in the protein sequence (non-synonymous) were considered in the analysis. To further verify the presence of the non-synonymous predicted SNPs, we retrieved and aligned the protein sequence of homologs where the SNPs were detected and confirmed visually the presence of the amino acid change. Non-synonymous SNPs were identified in 256 genes. We assessed the significance of our results performing 10 shuffling experiments (Supplementary Table S3B). We only found two SNPs in Experiment 1 and both were located in genes encoding for metabolic functions. Our test confirm that the SNPs correlation with CV strains is not random.

**FIGURE 6:**
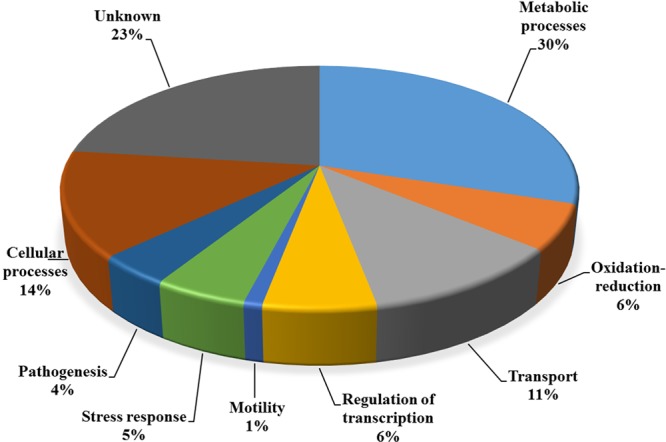
Functional distribution of non-synonymous SNPs conserved in CV strains. GO functional annotations were assigned to genes by biological annotation categories.

Gene annotation with non-synonymous SNPs distributed by biological process is presented in **Figure 6**. The main categories were unknown with 23% and metabolic processes with 30%. The rest of the category percentages were in the range of typical complete bacterial genomes. The list of genes with non-synonymous conserved SNPs can be found in Supplementary Table S5.

Analysis of previous studies on cool virulence and responses to salicylic acid (SA) indicated significant overlap of the SA regulon with our SNPs results. Thirty and sixteen percent of the genes annotated in the cellular processes and metabolic processes categories, respectively, were also identified previously as downregulated in presence of SA (Lowe-Power et al., 2016). In the latter category, most genes were related to the amino acids metabolic pathways. Thirty percent of the genes annotated in the oxidation-reduction category were also part of the SA regulon. Roughly, half of them were upregulated, including a catalase protein KatE that was previously identified (Bocsanczy et al., 2014a) as temperature dependent, and part of the HrpG regulon (Valls et al., 2006). KatE probably contributes to survival in the presence of an oxidative environment.

All the genes annotated in the motility category belong to the type IV twitching motility system. Twitching motility is associated with attachment and virulence in *R. solanacearum* (Kang et al., 2002). The twitching motility response is temperature dependent *in vitro* and *in planta* during the early stages of infection (Bocsanczy et al., 2012). PilC is a protein part of the structural twitching motility pilus, and PilB is an ATpase thought to power the pilus movement (Burdman et al., 2011), while PilJ has a chemotaxis domain and a homolog in *Pseudomonas aeruginosa* was required for functional pili (Mattick, 2002). SA repressed their gene expression (Lowe-Power et al., 2016). In the stress responses category 20% of the annotated genes were upregulated by SA (Lowe-Power et al., 2016). In addition, a secreted alkaline phosphatase was previously less abundant at 18°C (Bocsanczy et al., 2014a).

In the pathogenicity category, SA repressed 60% of the identified genes (Lowe-Power et al., 2016). We identified a polygalacturonase PehB that is part of the HrpG regulon (Valls et al., 2006). PehB contributes to disease in tomato (Huang and Allen, 2000). Three proteins in the eps operon or related to its function, also regulated by SA, were present in this category. Extracellular polysaccharides (EPS) contribute to virulence and stem colonization (Saile et al., 1997). Tek gene was identified in this category. It encodes a precursor protein which is exported and processed extracellularly to release a 28 kDA from its C-terminus (TeK). This is the single most abundant protein produced by *R. solanacearum* in culture (Denny et al., 1996). This protein abundance was temperature dependent in our proteomics previous study (Bocsanczy et al., 2014a). Although this protein is the most abundantly secreted during pathogenesis, and it is coregulated with EPS, it did not contribute to virulence or affect the levels of EPS during pathogenesis (Denny et al., 1996). A T3 effector AvrPphD also known as RipD was also identified. AvrPphD protein from *Pseudomonas syringae* pv. *phaseolicola* elicits non-host hypersensitive response (HR) on pea (*Pisum sativum*); it is also found in a great variety of *P. syringae* pathovars. As a virulence effector, it is less effective due to redundancy of effector functions (Arnold et al., 2001).

In the transcriptional regulators category 12.5% were repressed by SA (Lowe-Power et al., 2016) and 19% were downregulated at low temperatures (Meng et al., 2015). SolR and SolI are LuxR and Lux I homologs that regulate the production of two acyl-homoserine lactones (acyl-HSL). SolI expression is dependent on SolR and the presence of the acyl-HSL. Inactivation of SolI abolishes production of these acyl-HSL but does not affect the expression of virulence genes in culture or pathogenicity in tomato (Flavier et al., 1997). The acyl-HSL regulate the production of aidA in strain AW1 which is a phylotype IIA-seq 7 as K60 is, and SolR/SolI regulated expression of aidA in R3B2 strains (Meng et al., 2015). AidA is not present in the phylotype I and III strains but it is present in all phylotype II strains compared in this work including the NPLT strains K60, P597, P795 (CIP20), and CFBP2957. Interestingly a deletion of aidA in UW551 (CV strain) reduced the virulence of the strain in tomato significantly only at low temperature, while a deletion in solI did not (Meng et al., 2015). The presence of aidA in NPLT strains suggests that aidA might be involved in other functions besides cool virulence and/or that there could be more unknown regulatory pathways that might affect other genes related to cool virulence.

Another two component regulation pair of interest is phcS/phcR. This pair of genes with phcB conform an operon responsive to the quorum sensing signaling molecule 3-OH_PAME. PhcB is required for production of 3-OH_PAME and is autoregulated by it. 3-OH-PAME also regulates PhcS/R. PhcS/PhcR function together to reduce production of PhcA-regulated factors. The operon in absence of 3-OH-PAME (low density cells) regulates negatively production or activity of the global regulator PhcA at the top a complex hierarchical regulation chain of virulence and survival factors (Clough et al., 1997; Poussier et al., 2003). Additionally, two undescribed transcriptional regulators (Rsp0645 and Rsc1610) in this group were differentially regulated in GMI1000 and UW551 at low temperature (Meng et al., 2015). This fact makes them interesting candidates to be investigated.

Approximately 20–30% of genes with conserved SNPs in CV strains were related to responses to SA presence in the cellular processes, metabolic processes, and stress response categories. In motility and pathogenicity categories, the percentage was 100 and 60%, respectively. Additionally, several key transcriptional regulators such as SolR and Phcs/PhcS responsive to different secreted external molecules involved in quorum sensing have conserved SNPs in CV strains. This indicates that those genes directly or indirectly respond to environmental stimuli. It would be logical to think that these genes may respond also to temperature changes and it is possible they will respond differently in CV strains than in NPLT strains.

### Type 6 Secretion System

**FIGURE 7:**
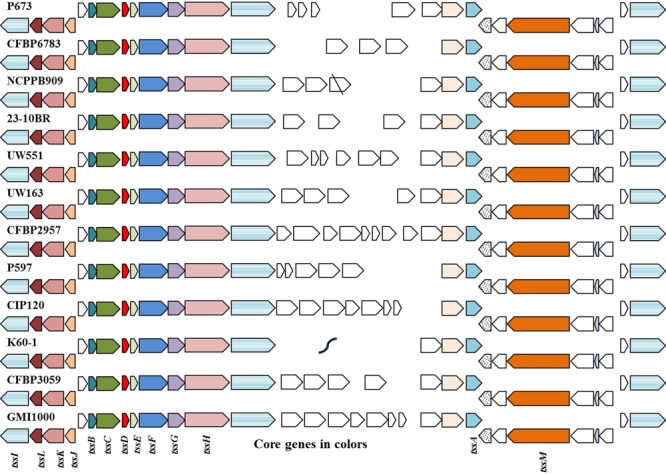
Type 6 secretion system cluster is highly conserved in *R. solanacearum* strains. T6SS clusters of 12 strains were aligned with MAUVE and orthologous genes were identified. Core genes and their orthologs are indicated in the bottom row by different colors and patterns.

The T6SS is a secretion system recently identified in gram-negative bacteria (Mougous et al., 2006; Schwarz et al., 2010a). It is highly conserved and ubiquitous in many gram-negative bacterial species (Das and Chaudhuri, 2003; Pukatzki et al., 2006). It has been associated with varied functions (Russell et al., 2014), including antimicrobial (Hood et al., 2010; MacIntyre et al., 2010), pathogenesis (Schell et al., 2007; Schwarz et al., 2010b), nodulation inhibitor (Bladergroen et al., 2003), and intra and inter-species competition (Schwarz et al., 2010b; Ma et al., 2014; Shyntum et al., 2015). T6SS is of interest in this comparison because T6SS its expression is different at low temperature between CV strains and NPLT ones (Bocsanczy et al., 2014b). Thirteen core genes have been identified in different bacterial species (Zheng and Leung, 2007), and have been classified in five different clades based on their sequence and structure in the cluster (Barret et al., 2013; Li et al., 2015). In our work, all *R. solanacearum* compared strains encode the 13 core genes and the cluster architecture is a type I subclade 4b. Type I are found in proteobacteria and are the most widespread type of T6SS clusters. As expected, the core genes are highly conserved among all the strains compared (**Figure 7**). For a complete list of T6SS core genes see Supplementary Table S6A. P799 T6SS cluster was not included in the comparison because the sequence of this region is incomplete in the draft genome. We added a closely related strain (CMR15) to show that the T6SS cluster conservation includes *R. solanacearum* Phylotype III.

Type 6 secretion system effectors are diverse, relatively unknown secreted proteins, thought to be translocated directly into the host cell cytosol. They can target eukaryotic cells (Pukatzki et al., 2007) or bacterial cells (Hood et al., 2010) depending of their function. We used a combination of Secret6 database searches (Li et al., 2015) and T6SS associated domains defined in the literature to scan the genomes for putative T6SS effectors. We identified 33 putative T6SS effector families (Supplementary Table S6B). VGR were the first effector domains to be identified in T6SS systems (Pukatzki et al., 2007). VGR is a protein that forms a complex with hemolysin coregulated protein (Hcp) to produce the cap structure thought to pierce the hosts cell membrane in an analogous process to virus injection (Pukatzki et al., 2007; Leiman et al., 2009; Silverman et al., 2013). VGR proteins can be found alone or fused with functional domains at their C-terminus. The modular structure of these effectors suggests that VGR in complex with Hcp may facilitate the secretion and translocation of the functional domains (Ho et al., 2014). We identified 21 VGR containing genes in the genomes of the compared strains (Supplementary Table S6B). Three families contained only the VGR domain and the rest were fusions of VGR, Rhs elements, and DUF2345 domain of unknown function. Rhs domains are also common in T6SS effectors, and they are composed of repeats associated with mobile elements and horizontal transfer (Jackson et al., 2009). It has been shown that Rhs proteins are T6SS secreted and mediate intercellular competition in *Dickeya dadantii* (Koskiniemi et al., 2013). We also identified four gene families containing a PAAR motif. Recently PAAR domains have been associated with T6SS effectors (Shneider et al., 2013). In our analysis, we found two families with only the PAAR motif domain and no conserved domain following it, however, there are VGR-Rhs proteins in close proximity. It is likely that the sequences were not properly annotated and there might be assembly errors due to the presence of the repetitive elements. One family contains the PAAR domain fused with Rhs repeats but no toxin module. Another family has a toxin module domain at the C-terminus. Other domains identified by similarity with known effectors in other bacterial species, included two peptidases, one with fused Rhs and discoidin domains, hemolysin translocator, a DUF1795 unknown conserved domain, two putative alpha/beta hydrolase and a lectin containing a DUF3274 of unknown function. The T6SS cluster contains two VGR proteins and four conserved proteins in all strains compared. None of the VGR proteins seem to be functional in all the strains (Supplementary Table S6A), suggesting that the VGR proteins may be interchangeable during secretion. Conserved proteins in the cluster included a putative lipoprotein, a glycosyl transferase, an OMPA-like cell envelope protein, and a hypothetical protein of unknown function. The fact that those proteins are conserved and complete in all the compared *R. solanacearum* strains, even in *Burkholderia* sp. strains, suggests that they might have an important function in the T6SS secretion. We could not identify any Tae4 superfamily in the genomes analyzed. The Tae4 superfamily is a newly identified form of toxin-antitoxin system associated with T6SS system, and has anti-microbial functions against other bacterial species (Russell et al., 2012). The lack of these pair of genes suggests that T6SS in *R. solanacearum* may not have antimicrobial function, but it may have a role in inter or intra-species competition. Although there is a high diversity in T6SS effectors, especially in the VGR domain containing families, we could identify only one putative operon that seems to be present exclusively in NPLT strains (Supplementary Table S6B). This operon (Rsp1137, Rsp1138, and Rsp1139 in GMI1000) is composed of a PAAR-Rhs toxin, and VGR domains, a hallmark of an “evolved” VGR T6 effector (Pukatzki et al., 2009), suggesting a role in translocation of toxins by T6SS. The results of our comparison have uncovered several potential functional T6 effectors that we are investigating in more detail.

### Type 3 Secretion System

**FIGURE 8:**
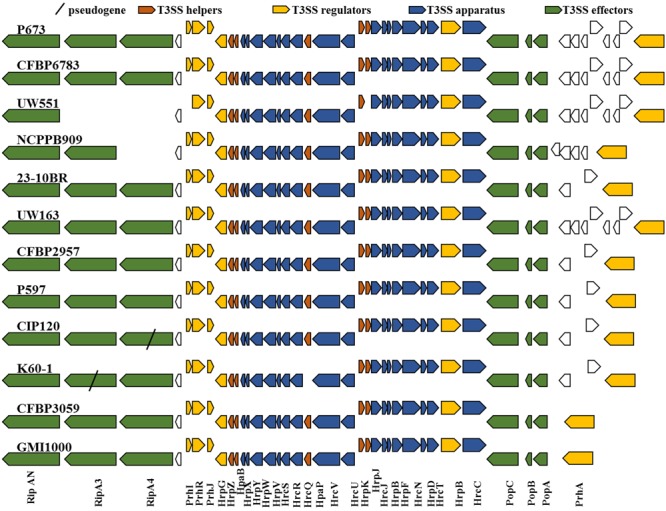
Type 3 secretion system cluster is highly conserved in *R. solanacearum* strains. T3SS cluster of twelve strains were aligned with MAUVE and homolog genes were identified. Red genes indicate T3SS helpers, blue indicate T3SS regulators, and red indicate T3SS effectors.

The T3SS cluster is highly conserved in all the different strains. All the core and T3SS accessory genes were present in all the strains compared (**Figure 8**). This comparison confirms previous observations of high conservation in the T3 cluster genes (Van Gijsegem et al., 1993).

Type 3 secretion system effectors were predicted using the “ScanYourGenome” software and RalstoT3E database (Peeters et al., 2013). “ScanYourGenome” software predicts ORFs based on similarity to known T3E in other bacterial species, presence of HrpII boxes (to predict regulation by HrpB), and presence of T3SS-dependent export pattern (Petnicki-Ocwieja et al., 2002). The software classifies the predicted T3E by similarity with orthologous families defined in the RalstoT3E database and assigns a unified nomenclature: Ralstonia Injected Proteins (Rip). Predicted T3E were further inspected for homology in the group of compared strains, and pseudogenes status verified manually. Frameshifts were assumed as functional in WGS genomes, since a frameshift may be due to errors in sequencing and assembly. The repertoires vary from one strain to the other. We identified 17 core effectors (complete in all the compared strains) of which 14 coincide with the core effectors identified in Peeter’s T3E repertoires (Peeters et al., 2013) (Supplementary Table S7). Analogous to Peeter’s analysis looking for a correlation between T3E repertoires and host range, we did not find a correlation of T3E repertoire and cool virulence as there was not one putative effector that would be present in all the CV strains compared and absent in the NPLT ones or vice-versa. These results suggest that the T3 effectors are not involved in the CV phenotype.

This genomic comparative analysis of *R. solanacearum* strains complements previous works on transcriptomics and proteomics in the search for temperature related virulence factors. Currently identified CV strains are restricted to phylotype IIB clade sequevar 1 and 4, supporting the idea that the phenotype arose before the split of sequevars. A high percentage of transcriptional regulators and a practical absence of known virulence related genes that are present only in CV strains supports the hypothesis that regulation of virulence genes could explain the difference in virulence at low temperatures between the groups compared. This study as previous ones on cool virulence factors supports the hypothesis that cool virulence depends on multiple genes and multiple regulation pathways. This comparison contributes to find new possible connections of temperature dependent virulence to the described complex regulatory system involving quorum-sensing, phenotype conversion (phcA), acyl-HSL production and responses to SA. It also identified seven transcriptional regulators with high likelihood to be involved in regulation of cool virulence. This comparative work has also identified for the first time, putative T6 effectors that could be involved in differential fitness or aggressiveness of the strains compared.

## Author Contributions

AB designed the experiments, performed the assemblies, comparative analysis and experiments, and wrote the manuscript. JH-T supported the bioinformatics work for the comparative analysis and reviewed the manuscript. DN conceived the work, supervised, coordinated all activities and provided critical review to the manuscript.

## Conflict of Interest Statement

The authors declare that the research was conducted in the absence of any commercial or financial relationships that could be construed as a potential conflict of interest.

## Footnotes

1 http://www.geneious.com

2 https://www.genoscope.cns.fr/agc/microscope/home/index.php

## Abbreviations

CDS: coding sequences
COG: Clusters of Orthologous Genes
CV: cool virulent
EMBL: European Molecular Biology Laboratory
EPS: exopolysaccharides
NCBI: National Center for Biotechnology Information
NPLT: non-pathogenic at low temperatures
PGAP: prokaryotic genome annotation pipeline
R3B2: race 3 biovar 2
SNP: single nucleotide polymorphisms
T3SS: type 3 secretion system
T6SS: type 6 secretion system
