# Supplementary material for: Comparative Genomics of Ralstonia solanacearum Identifies Candidate Genes Associated with Cool Virulence
Source: Front Plant Sci. 2017 Sep 13;8:1565. doi: 10.3389/fpls.2017.01565 (PMC5601409; doi:10.3389/fpls.2017.01565)
Supplement: Supplementary file 2 [file Table_2.DOCX]

| **Strain** | **P597** | **P795** | **P797** | **P799** |
| --- | --- | --- | --- | --- |
| **Assembled Reads statistics** |  |  |  |  |
| Total # Read pairs | 4938638 | 4985729 | 4518903 | 3720193 |
| # Reads assembled | 4819774 | 4814086 | 4398358 | 3542921 |
| # Unused Reads | 118864 | 171643 | 120545 | 177272 |
| Average quality (Phred) score | 36 | 36 | 36 | 35.5 |
| **Assembly totals** |  |  |  |  |
| # all Contigs | 20385 | 3480 | 1797 | 2873 |
| # Contigs>1K | 534 | 135 | 163 | 373 |
| # Contig >=N50 | 177 | 26 | 29 | 72 |
| N50 average length (bp) | 10310 | 79115 | 57264 | 22902 |
| Average coverage | 252X | 178X | 202X | 140X |
| Average contig length | 411 | 1788 | 3233 | 2014 |
| Length Sum (bp) all contigs | 8383548 | 6224058 | 5809993 | 5786475 |
| **Scaffold assembly** |  |  |  |  |
| # contigs mapped on pseudomolecule | 530 | 145 | 178 | 399 |
| Length of mapped contigs | 5653308 | 5685940 | 5574885 | 5436189 |
| Length of unmapped contigs all contigs | 2730240 | 538118 | 235108 | 350286 |

**Supplementary Table S2.** Assembly statistics for the *R. solanacearum* genomes sequenced for this work.
