# Supplementary material for: Comparative Genomics of Ralstonia solanacearum Identifies Candidate Genes Associated with Cool Virulence
Source: Front Plant Sci. 2017 Sep 13;8:1565. doi: 10.3389/fpls.2017.01565 (PMC5601409; doi:10.3389/fpls.2017.01565)
Supplement: Supplementary file 8 [file Presentation_1.PPTX]

## Slide 1
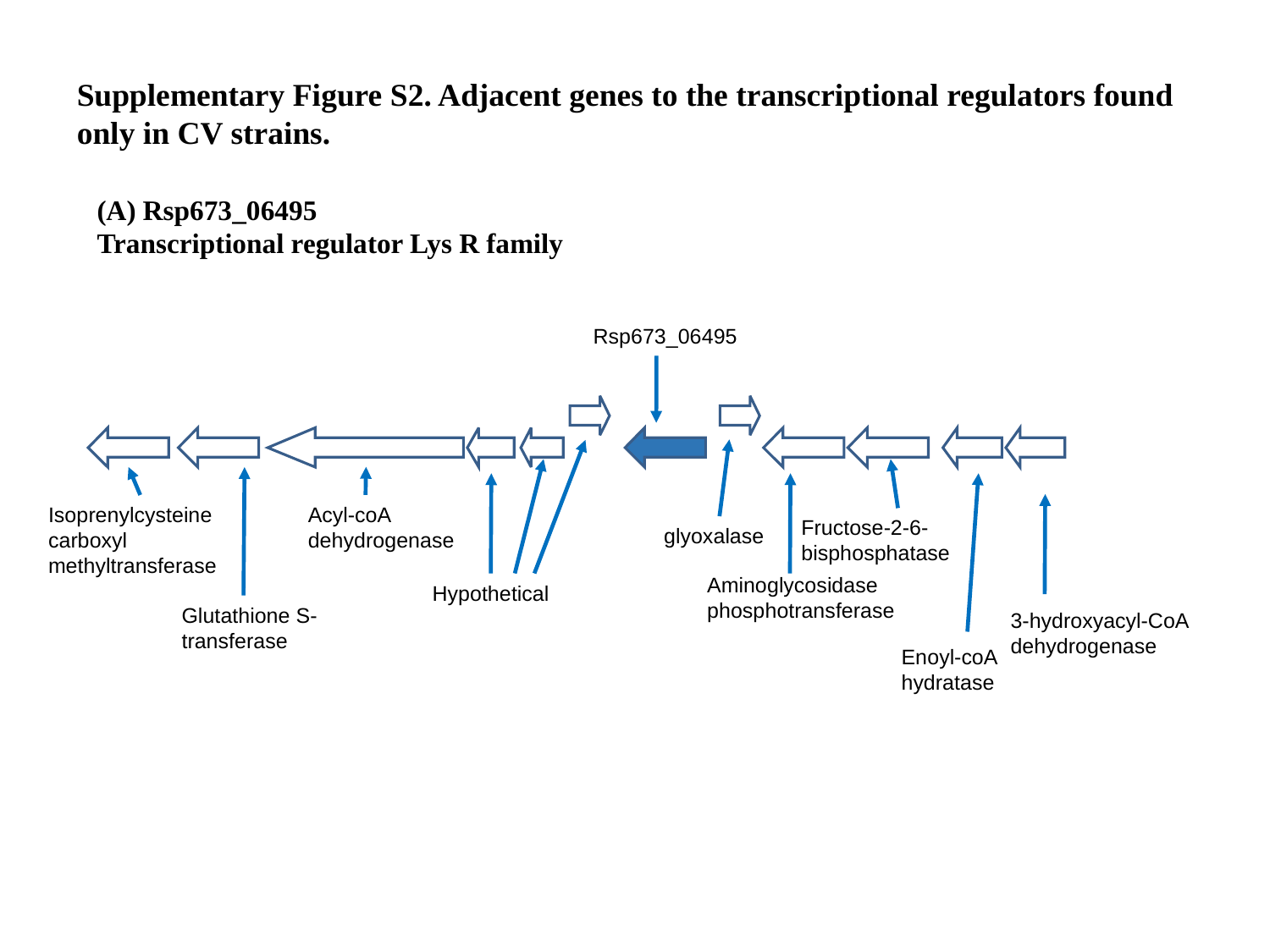

Supplementary Figure S2. Adjacent genes to the transcriptional regulators found only in CV strains.
(A) Rsp673_06495
Transcriptional regulator Lys R family
Rsp673_06495
Isoprenylcysteine carboxyl methyltransferase
Acyl-coA dehydrogenase
Fructose-2-6-bisphosphatase
glyoxalase
Aminoglycosidase phosphotransferase
Hypothetical
Glutathione S-transferase
3-hydroxyacyl-CoA dehydrogenase
Enoyl-coA hydratase

## Slide 2
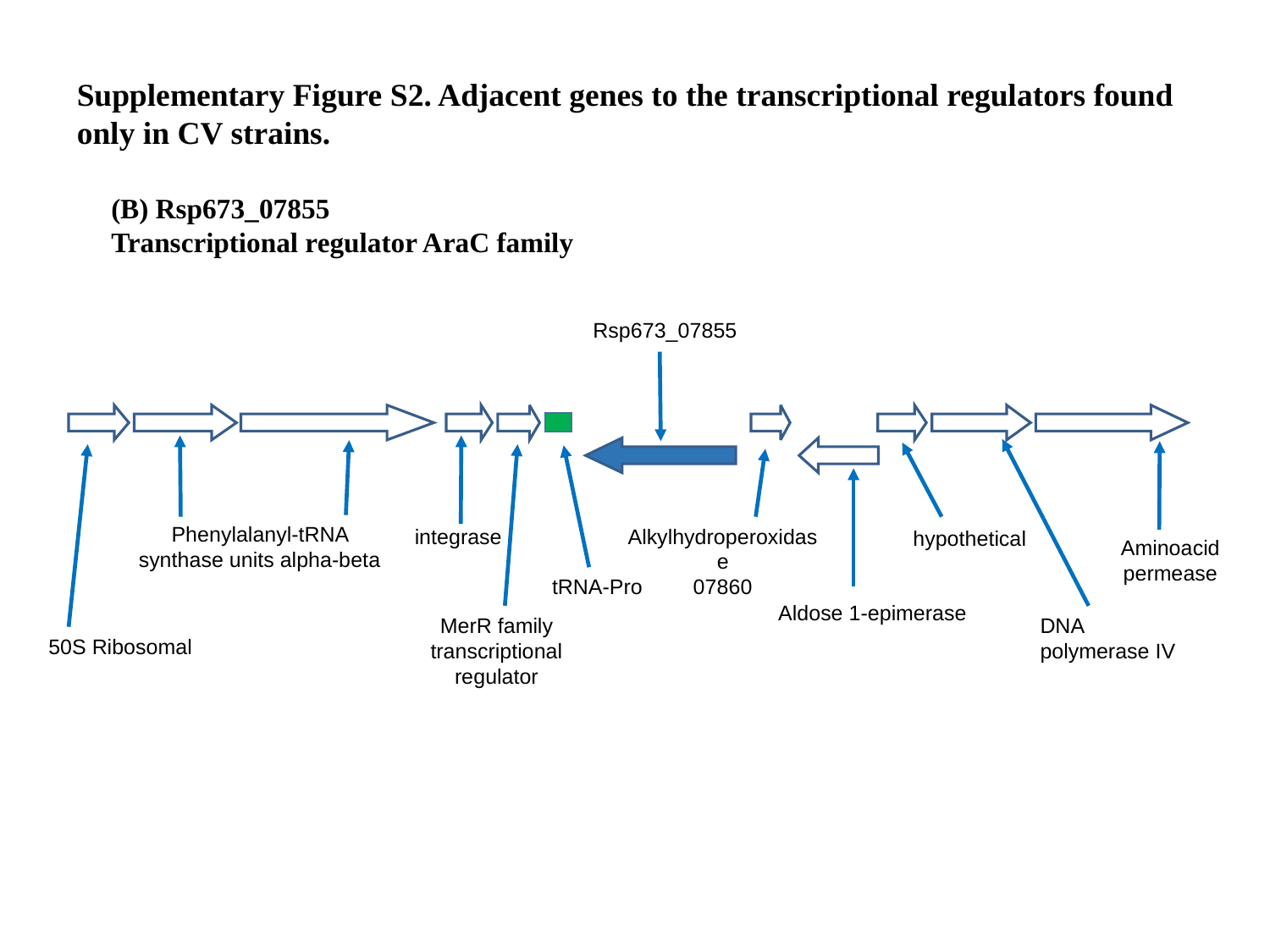

Supplementary Figure S2. Adjacent genes to the transcriptional regulators found only in CV strains.
(B) Rsp673_07855
Transcriptional regulator AraC family
Rsp673_07855
Phenylalanyl-tRNA synthase units alpha-beta
integrase
Alkylhydroperoxidase
07860
hypothetical
Aminoacid permease
tRNA-Pro
Aldose 1-epimerase
MerR family transcriptional regulator
DNA polymerase IV
50S Ribosomal

## Slide 3
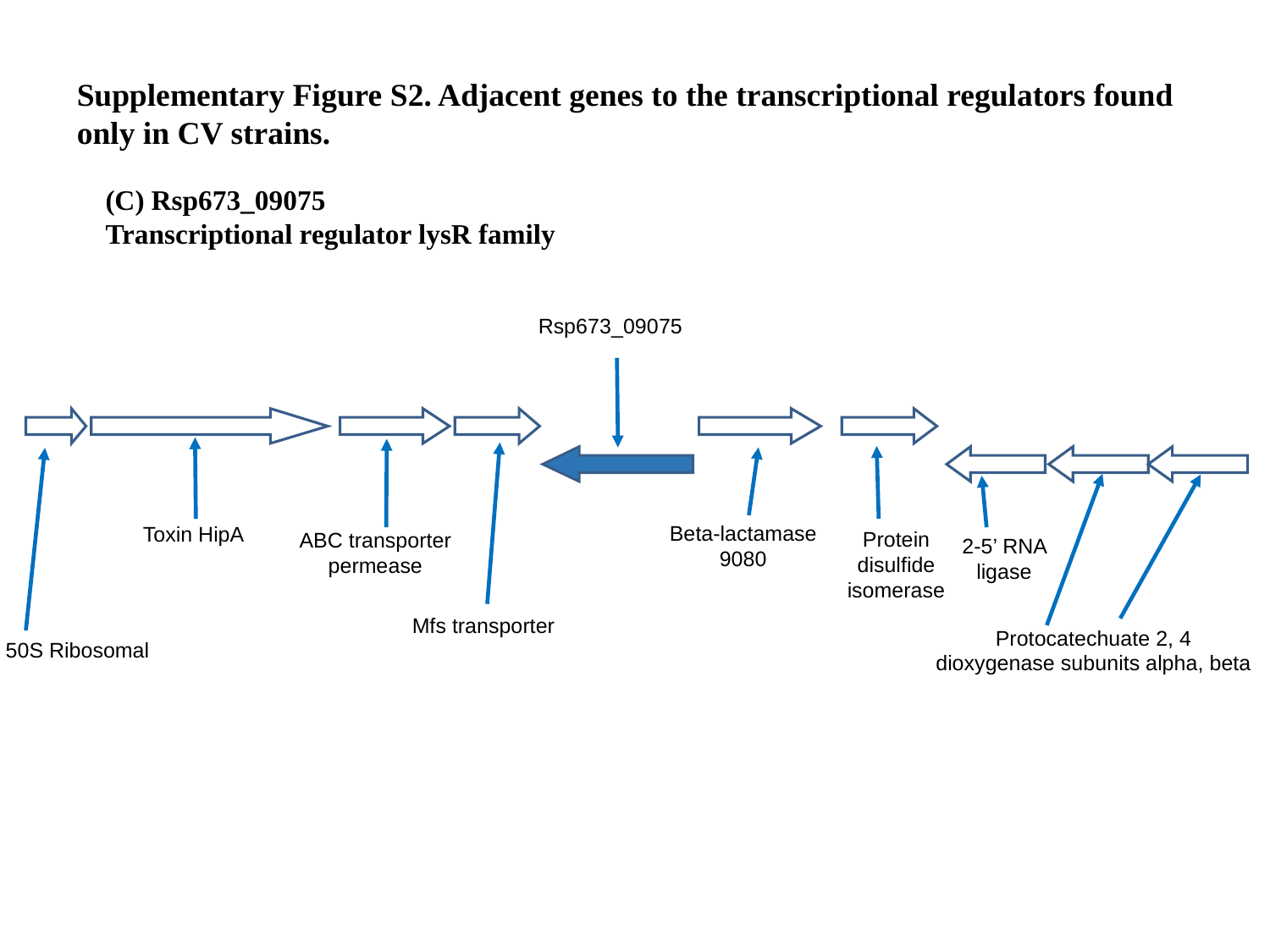

Supplementary Figure S2. Adjacent genes to the transcriptional regulators found only in CV strains.
(C) Rsp673_09075
Transcriptional regulator lysR family
Rsp673_09075
Beta-lactamase
9080
Toxin HipA
Protein disulfide isomerase
ABC transporter permease
2-5’ RNA ligase
Mfs transporter
Protocatechuate 2, 4 dioxygenase subunits alpha, beta
50S Ribosomal

## Slide 4
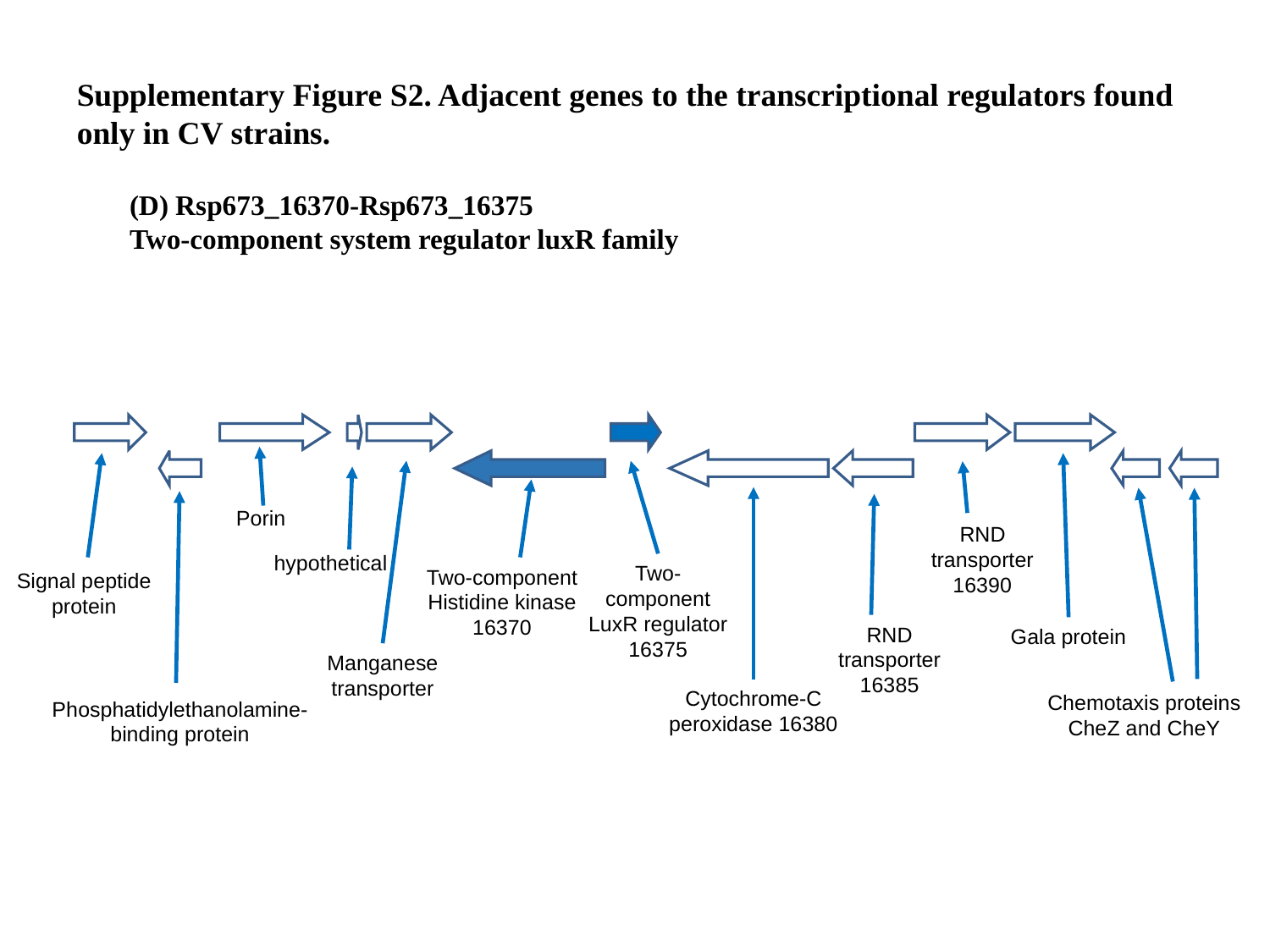

Supplementary Figure S2. Adjacent genes to the transcriptional regulators found only in CV strains.
(D) Rsp673_16370-Rsp673_16375
Two-component system regulator luxR family
Porin
RND transporter
16390
hypothetical
Two-component LuxR regulator 16375
Two-component Histidine kinase
16370
Signal peptide protein
RND transporter
16385
Gala protein
Manganese transporter
Cytochrome-C peroxidase 16380
Chemotaxis proteins CheZ and CheY
Phosphatidylethanolamine-binding protein

## Slide 5
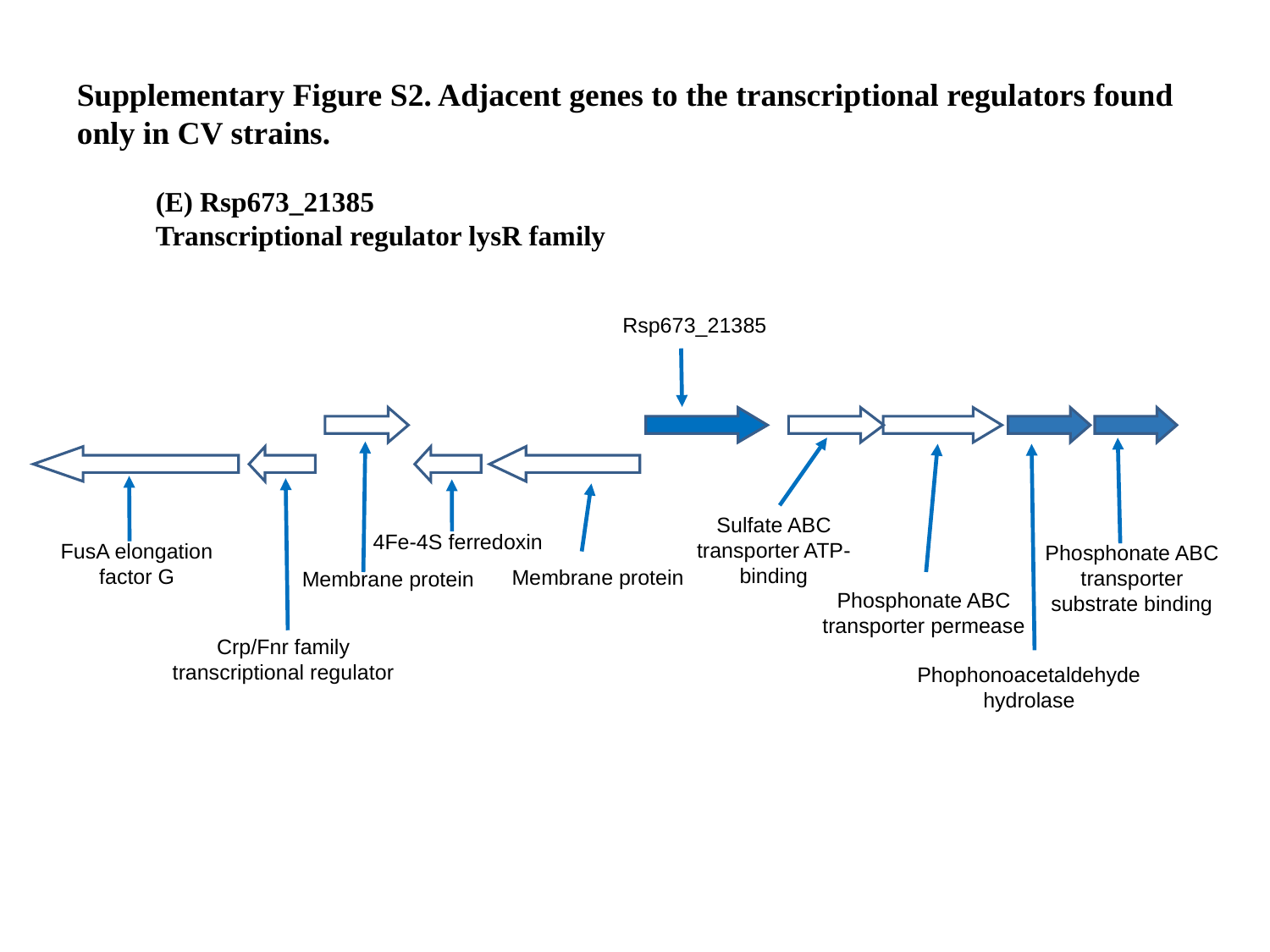

Supplementary Figure S2. Adjacent genes to the transcriptional regulators found only in CV strains.
(E) Rsp673_21385
Transcriptional regulator lysR family
Rsp673_21385
Sulfate ABC transporter ATP-binding
4Fe-4S ferredoxin
FusA elongation factor G
Phosphonate ABC transporter substrate binding
Membrane protein
Membrane protein
Phosphonate ABC transporter permease
Crp/Fnr family transcriptional regulator
Phophonoacetaldehyde hydrolase

## Slide 6
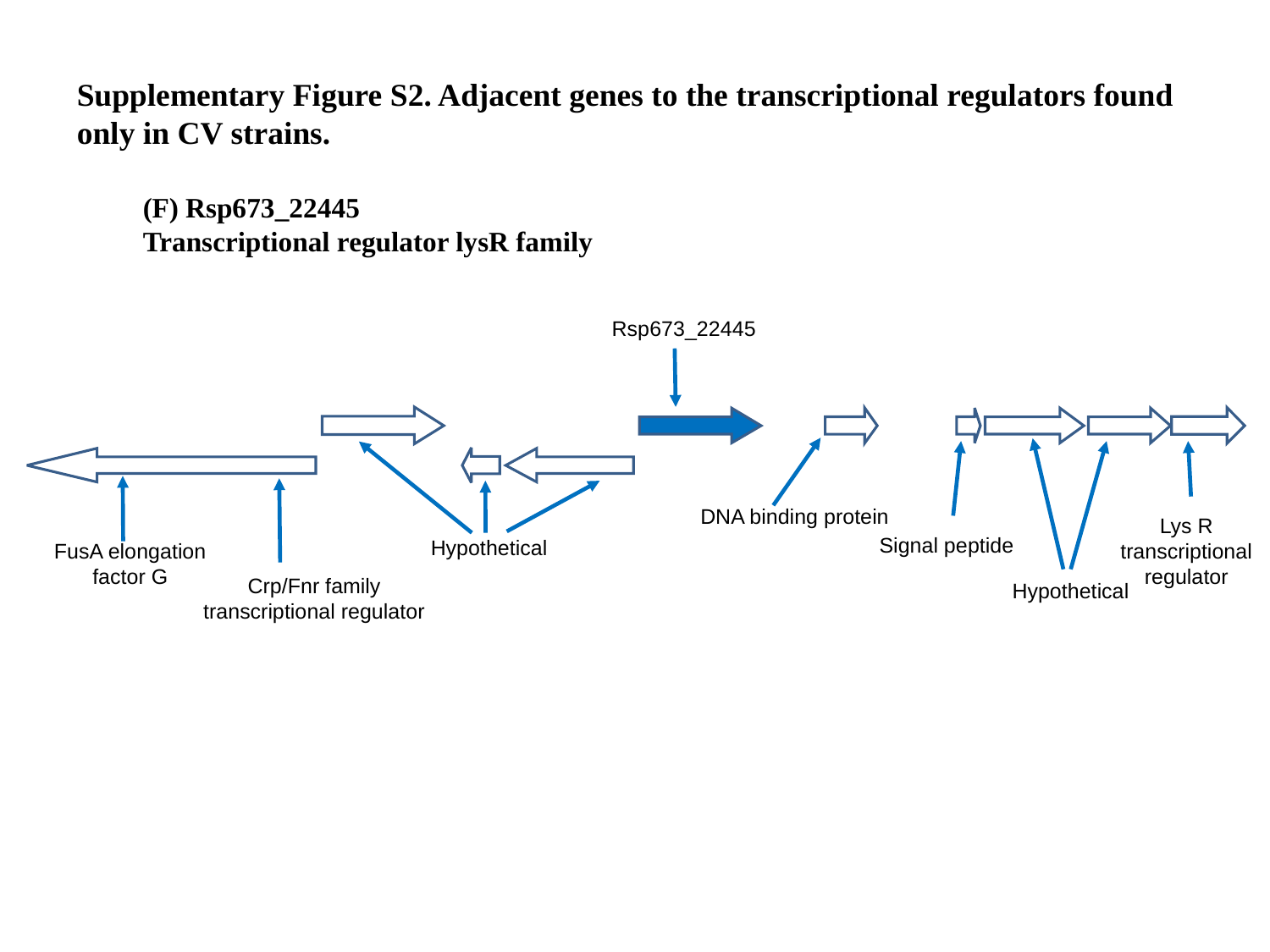

Supplementary Figure S2. Adjacent genes to the transcriptional regulators found only in CV strains.
(F) Rsp673_22445
Transcriptional regulator lysR family
Rsp673_22445
DNA binding protein
Lys R transcriptional regulator
Signal peptide
Hypothetical
FusA elongation factor G
Crp/Fnr family transcriptional regulator
Hypothetical

## Slide 7
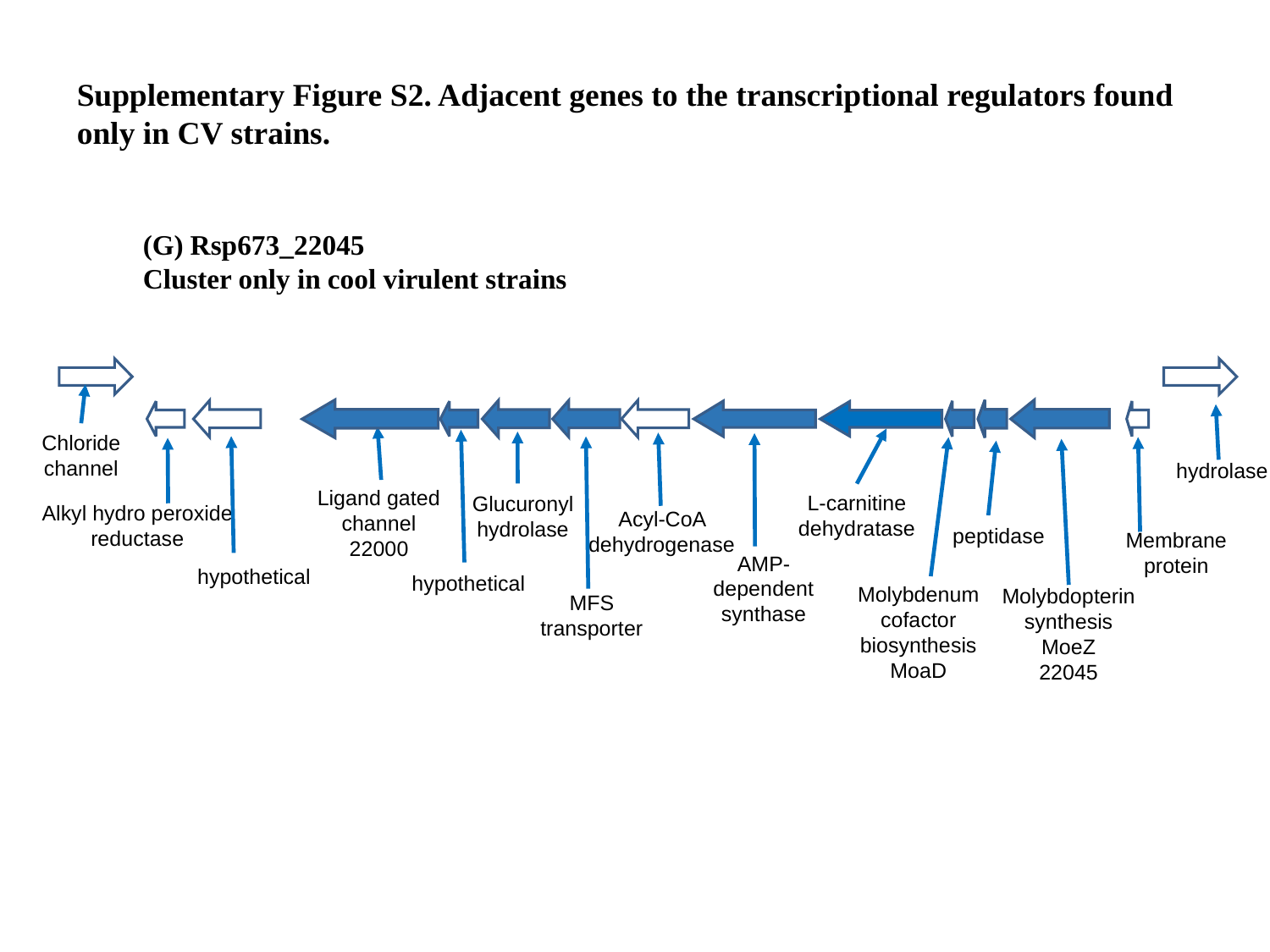

Supplementary Figure S2. Adjacent genes to the transcriptional regulators found only in CV strains.
(G) Rsp673_22045
Cluster only in cool virulent strains
Chloride channel
hydrolase
Ligand gated channel
22000
L-carnitine dehydratase
Glucuronyl hydrolase
Alkyl hydro peroxide reductase
Acyl-CoA dehydrogenase
peptidase
Membrane protein
AMP-dependent synthase
hypothetical
hypothetical
Molybdenum cofactor biosynthesis MoaD
Molybdopterin synthesis MoeZ
22045
MFS transporter
