# Supplementary figures and images for: Comparative Genomics of Ralstonia solanacearum Identifies Candidate Genes Associated with Cool Virulence
Source: Front Plant Sci. 2017 Sep 13;8:1565. doi: 10.3389/fpls.2017.01565 (PMC5601409; doi:10.3389/fpls.2017.01565)

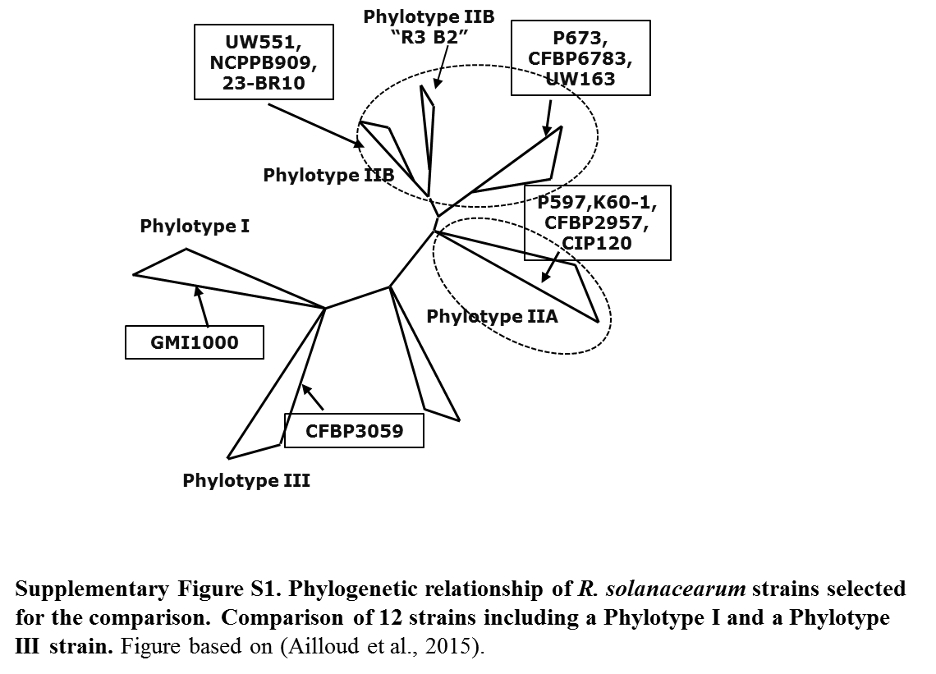

Supplement: Supplementary file 9 [file Image_1.JPEG]

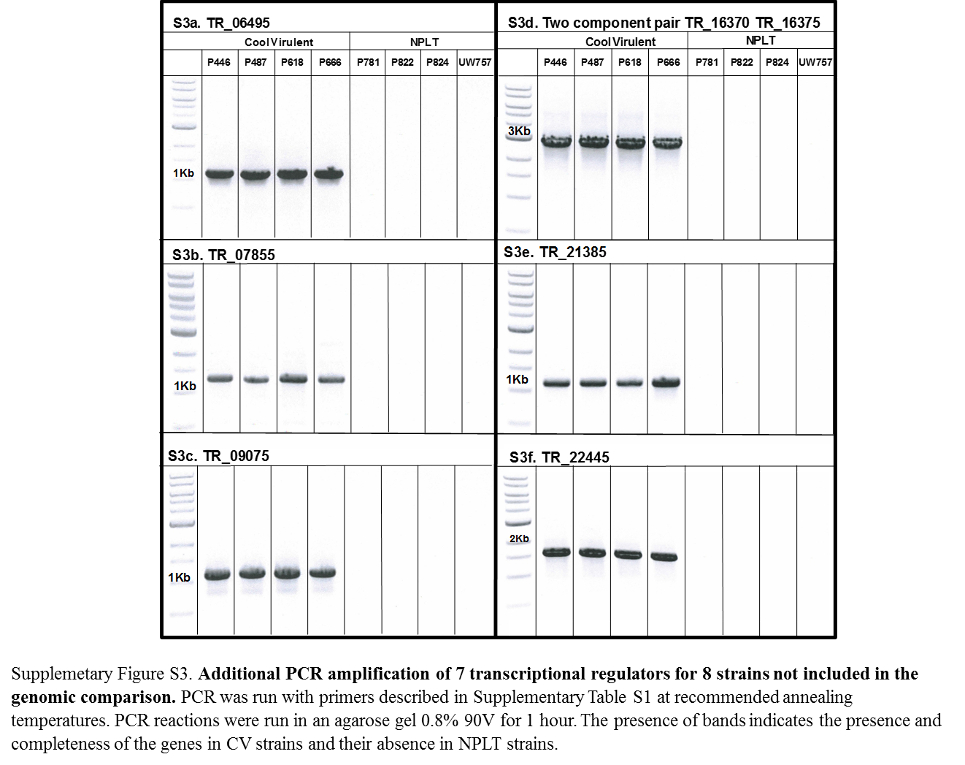

Supplement: Supplementary file 10 [file Image_3.JPEG]
